# Strong Inhibition of Xenografted Tumor Growth by Low-Level Doses of [^32^P]ATP

**DOI:** 10.18632/oncotarget.289

**Published:** 2011-06-05

**Authors:** Yulan Cheng, Jian Yang, Rachana Agarwal, Gilbert M. Green, Ron C. Mease, Martin G. Pomper, Stephen J. Meltzer, John M. Abraham

**Affiliations:** ^1^ Department of Medicine, Johns Hopkins School of Medicine, Baltimore, MD 21287; ^2^ Department of Radiology, Johns Hopkins School of Medicine, Baltimore, MD 21287

**Keywords:** xenografts, inhibition, nude mice

## Abstract

The ability of a potential human anti-cancer therapeutic agent to inhibit the growth of xenografted tumors in nude mice has been an established and accepted testing method for several decades. The current report shows that a single, low-level intravenous dose of [^32^P]ATP significantly inhibits the growth of established xenografted tumors in nude mice. This inhibitory effect becomes appreciable very rapidly, within only five days post-injection and the low dose demonstrates little or no toxicity in the mice. Surprisingly, a narrow dose window of optimum effectiveness is seen, whereby either decreasing or increasing the [^32^P]ATP dose results in far less growth inhibition. Thus, the intravenous systemic injection of [^32^P]ATP may represent a simple, potent method to target and inhibit primary human tumors and malignant lesions.

## INTRODUCTION

Radioisotopes that emit beta particles can effectively kill target tumor cells. This therapeutic effect is exemplified by the ability of ^131^I to home naturally to thyroid tissue, resulting in very efficient treatment of thyroid cancer and Graves’ Disease. Two FDA-approved radioimmunotherapeutics utilizing antibodies against the CD20 cell surface marker, ^131^I-Bexxar and ^90^Y-Zevalin, are currently approved for the treatment of non-Hodgkin’s lymphoma [1,2]. In addition, neuroendocrine tumors can be treated with ^90^Y-radiolabeled somatostatin. The maximum beta energy of electrons emitted by [^32^P]ATP, the isotope utilized in the current experiments, lies intermediate between that of ^90^Yttrium and ^131^Iodine and has a path length range of up to 5 mm. in tissues. This range means that each electron penetrates hundreds to thousands of cells, resulting in a “bystander effect” that amplifies the killing power of each ^32^P atom in or near a tumor. The longer half-life of ^32^P compared to these other two isotopes represents an advantage, because the level of radioactivity present in the tumor will not diminish as rapidly due to natural decay.

For decades, evaluation of potential anticancer therapeutics has relied on their capacity to inhibit the growth of xenografted tumors in nude mice [3,4]. Successful drugs have included antibodies directed against cell surface molecules (e.g., Rituxan), or against broader targets, such as inhibition of the establishment of the tumor microenvironment (by anti-VEGF, e.g., Avastin) or the targeting of rapidly growing cells (by cisplatin) [5]. In general, smaller anti-cancer therapeutic molecules offer better tumor penetration as well as lower immunogenicity. The current report establishes that a single, low-dose intravenous injection of [^32^P]ATP rapidly and effectively inhibits the growth of xenografted tumors in nude mice. These tumors are already vascularized and established before the dose is administered, and a narrow dose window of effectiveness is seen.

## RESULTS

### Inhibition of xenografted tumor growth

**Figure 1:**
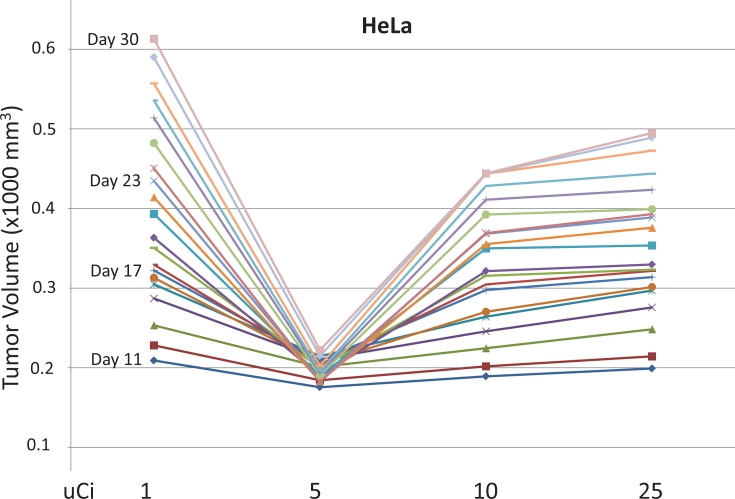
Inhibition of xenografted tumor growth HeLa cells (right rear flank) were used to establish xenografted tumors and were injected subcutaneously on Day 1. One of four different amounts of [^32^P]ATP was injected intravenously on Day 9. Means of the daily tumor volume measurement from Day 11 through Day 30 demonstrated a strong but narrow dose window of tumor growth inhibition centered at 5 μCi. Tumor volumes are 1000 x mm^3^.

Ten week old nude mice received subcutaneous injections of human colon adenocarcinoma cell line HCT116 in the left rear flank and human cervical carcinoma cell line HeLa in the right rear flank. By Day 9 the xenografted tumors were established and vascularized, at which time groups of five to six mice received a single intravenous tail vein injection of 1, 5, 10 or 25 μCi of [^32^P]ATP. Figure 1 shows the measured tumor volumes of the HeLa-derived xenografted tumors beginning at Day 11, two days after the radioactive dose was administered. Within one week of the [^32^P]ATP injection (by Day 16), a strong inhibition of xenografted tumor growth occurred at the 5 μCi dose level. This inhibition of tumor growth strengthened over the next two weeks, and a narrow optimal dose window was observed, which centered at 5 μCi. None of the mice used in any experiment in this report ever demonstrated any sign of radiation toxicity (*i.e.*, weight loss or by any diminution of mobility or activity).

**Figure 2:**
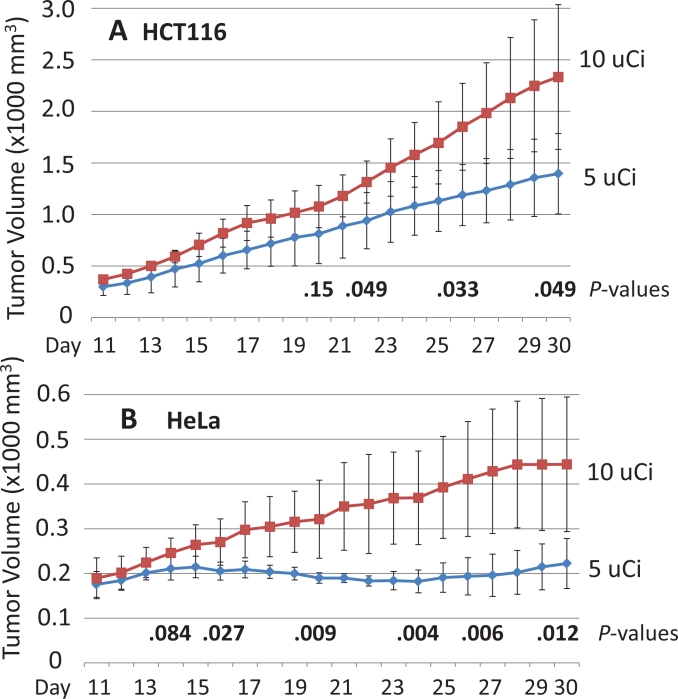
The human colon cancer cell line HCT116 (left rear flank) or HeLa cells (right rear flank) were used to establish xenografted tumors and were injected subcutaneously on Day 1 One of four different amounts of [^32^P]ATP was injected intravenously on Day 9. Significant tumor growth inhibition by comparing 5 μCi versus 10 μCi doses against HCT116-derived xenografted tumors (A) or by 5 μCi versus 10 μCi doses against HeLa-derived tumors (B) was detected as soon as Day 16, seven days after the [^32^P]ATP injections. The means and plus/minus one standard deviation are shown and the bold numbers are *P* values determined by the two-sided student’s t test. Tumor volumes are 1000 x mm^3^.

In our experiments, HCT116-derived tumors grew approximately five times faster than did HeLa-derived tumors. Figure 2A shows that the 5 μCi [^32^P]ATP dose was significantly superior to the 10 μCi dose in inhibiting tumor growth from HCT116-derived tumors, although this inhibition required one extra week versus the first appearance of inhibition in HeLa-derived tumors. Figure 2B reveals that the average volume of the HeLa-derived tumors in the mice receiving the 5 μCi soon plateaued, and then began to decrease by ten days [^32^P]ATP post-injection (at Day 19). Surprisingly, mice that received only a twice-greater dose (10 μCi) demonstrated greatly decreased tumor growth inhibition, and the tumor volume difference between mice receiving the 5 μCi versus the 10 μCi dose became extremely significant over the ensuing two weeks (*P*<0.004).

### Confirmation of growth inhibition using a second HeLa cell isolate

**Figure 3:**
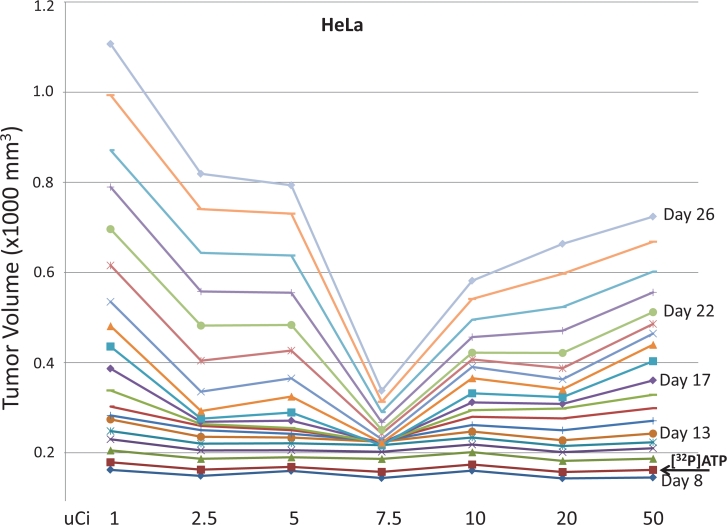
Inhibition of xenografted tumor growth Nude mice were injected subcutaneously with HeLa cells to establish xenografted tumors on Day 1 and one of seven different amounts of [^32^P]ATP was injected intravenously on Day 9 (arrow). Means of the daily tumor volume measurement from Day 8 through Day 26 demonstrated a narrow dose window of tumor growth inhibition centered at 7.5 μCi. Tumor volumes are 1000 x mm^3^.

**Figure 4:**
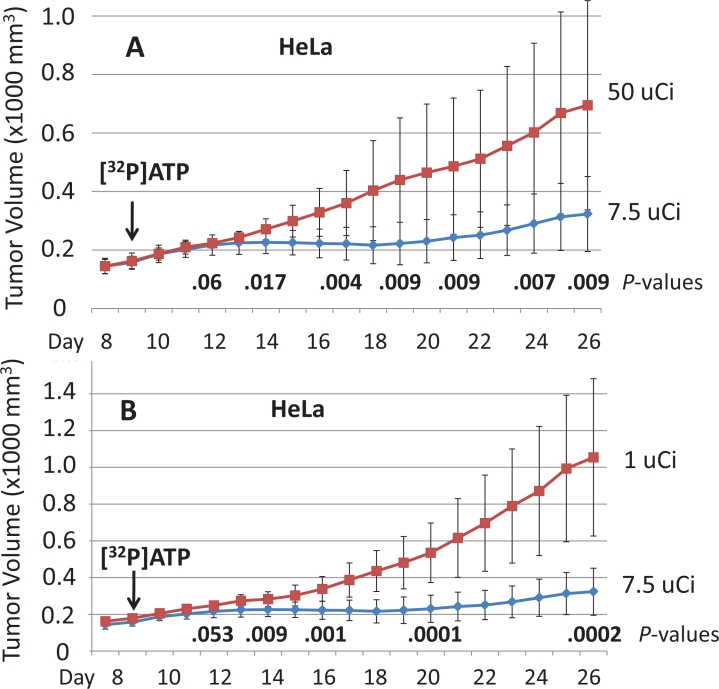
Nude mice were injected subcutaneously with HeLa cells to establish xenografted tumors on Day 1 and one of different amounts of [32P]ATP was injected intravenously on Day 9 Significant tumor growth inhibition by 7.5 μCi versus 50 μCi (A) or by 7.5 μCi versus 1 μCi (B) was detected as soon as Day 14, five days after the [^32^P]ATP injections. The means and plus/minus one standard deviation are shown and the bold numbers are *P* values determined by the two-sided student’s t test. Tumor volumes are 1000 x mm^3^.

The HeLa cell isolate used to establish the tumors shown in Figures 1 and 2 had been grown continuously in cell culture for more than four years. Cell lines grown in cell culture for extended periods can alter their genotypes and phenotypic attributes. Therefore, to test and establish the most reproducible system possible, a different isolate of HeLa was purchased from ATCC, grown in cell culture for two weeks, and used immediately to establish the HeLa-derived tumors in Figures 3 and 4. Figure 3 shows daily tumor volumes measured from multiple groups of nude mice that had been injected with HeLa cells subcutaneously on both the left rear and right rear flanks on Day 1, followed by one of seven different doses of intravenous [^32^P]ATP injected intravenously on Day 9. A narrow, but clearly defined dose window is evident, with maximum tumor growth inhibition occurring at 7.5 μCi. Significantly less inhibition was seen with [^32^P]ATP doses only 2.5 μCi above and below this optimal level. Figures 4A and 4B reveal the speed and magnitude at which these growth rate differences emerge in 7.5 μCi versus 50 μCi or 1 μCi doses, respectively.

## DISCUSSION

The mechanism for the reproducible finding of low-dose [^32^P]ATP-induced tumor growth inhibition is not understood at this time. We speculate that this effect may be due to a combination of factors, such as the direct cell-killing ability of the radioisotope, injury to the B cells and natural killer cells that are present and active in nude mice, and the phenomenon of low-dose radiation hypersensitivity of certain cells [6]. Inorganic elemental ^32^P has been used clinically to treat very high levels of platelets or red blood cells, thrombocythemia and polycythemia vera, respectively, and up to 15 mCi of inorganic sodium phosphate ^32^P can be administered to patients with chronic leukemia [7]. However, organic ^32^P, specifically in the form of [^32^P]ATP, has to our knowledge, not been reported in a therapeutic context. The levels of [^32^P]ATP used in nude mice in this report, correspond on a weight basis, to comparable levels of inorganic ^32^P used in accepted human treatments. In addition, current clinical trials utilize inorganic forms of ^32^P and nanotechnology techniques to attempt to deliver the radioisotope to target cells and cancers.

Previous studies found extracellular ATP at concentrations in excess of 100 μM in xenografted mouse tumors, but undetectable levels in healthy tissues [8]. Exogenously administered [^32^P]ATP may constitute a naturally targeted anticancer therapeutic agent and may involve cancer-related inflammation and the tumor microenvironment [9]. Additional advantages of the [^32^P]ATP molecule include the facts that 1) it is readily accessible and inexpensive, 2) it is a pure beta particle emitter with an average beta energy greater than ^131^I, 3) it is easy to handle, and 4) it has a long a half-life greater than two weeks. In addition to producing humanized antibodies that diminish the patient’s immune response against the Ab-based anti-cancer agent, bioengineering has also yielded smaller antibody molecules to enhance tumor penetration. Classically, anti-cancer molecules of smaller sizes often demonstrate better tumor penetration [10]. With a molecular weight of approximately 508 Daltons, [^32^P]ATP is extremely small relative to almost any other proposed or active anti-cancer agents, with the exception of ^131^I. [^32^P]ATP appears to take advantage of a natural tumor-homing ability and a unique requirement for exogenous ATP by the tumor microenvironment for its establishment and growth. Finally, the intravenous systemic injection of [^32^P]ATP may represent a simple, yet potent method to target and inhibit primary human tumors and malignant lesions.

## MATERIAL and METHODS

### Cell lines used to establish nude mouse xenografted tumors

All cell lines were purchased from ATCC and grown in MEM with 10% fetal bovine serum and Pen/Strep antibiotics. The HCT116 cell line used to establish the xenografted tumors shown in Figure 2A was grown in cell culture for approximately six months before injection, and the HeLa cell line shown in Figure 1 and in Figure 2B was grown in cell culture for approximately four years before injection. In an effort to maximize reproducibility in all experiments, the HeLa cell line used to establish the xenografted tumors in Figure 3 and in Figure 4 was purchased from ATCC, grown in cell culture for two weeks and then injected subcutaneously. The identities of all cell lines were confirmed using a short tandem repeat assay (Promega PowerPlex). The two HeLa cell lines were confirmed to be HeLa cells, although they were different isolate clones obtained from ATCC, as was expected.

### Establishment of xenografted tumors and injection of radioisotope in athymic nude mice

2 X 10^6^ cells in Matrigel (50%)/ 1 X PBS (50%) in 0.2 ml were injected subcutaneously into the left rear or right rear flank of female nude mice (Charles River, Boston) that were nine to ten weeks of age. [^32^P]ATP (3000 Ci/mmol) was purchased from Perkin Elmer and the indicated dose was injected in 0.1 ml of 1 X HBSS via the tail vein. Mouse weight and tumor volume were measured daily. The width (W) and length (L) of the tumor were measured using a digital caliper and the volume determined using the formula V = ½(L X W^2^). Five or six mice were used in each group. No mice in any experiments shown in Figures 1, 2, 3, or 4 showed any signs of toxicity as determined by significant differences in weight or by general activity levels. The xenografted tumor growth patterns seen at the 7.5 μCi [^32^P]ATP dose in Figures 3 and 4 and at the 5 μCi [^32^P]ATP dose seen in Figures 1 and 2 have xenografted tumors established and vascularized, with a steady tumor volume increase until the [^32^P]ATP is injected. At the optimal dose, after a few days, the growth patterns plateau and the tumor volume slightly declines, beginning as soon as five days post-injection. Approximately two weeks after [^32^P]ATP injection, a slight increase in tumor volume is observed at the optimal dose levels.
